# Role of Stat3 in Skin Carcinogenesis: Insights Gained from Relevant Mouse Models

**DOI:** 10.1155/2013/684050

**Published:** 2013-03-21

**Authors:** Everardo Macias, Dharanija Rao, John DiGiovanni

**Affiliations:** ^1^Division of Pharmacology and Toxicology, College of Pharmacy, The University of Texas at Austin, Austin, TX 78723, USA; ^2^Department of Nutritional Sciences, College of Natural Sciences, The University of Texas at Austin, Austin, TX 78723, USA; ^3^Dell Pediatric Research Institute, The University of Texas at Austin, 1400 Barbara Jordan Boulevard, Austin, TX 78723, USA

## Abstract

Signal transducer and activator of transcription 3 (Stat3) is a cytoplasmic protein that is activated in response to cytokines and growth factors and acts as a transcription factor. Stat3 plays critical roles in various biological activities including cell proliferation, migration, and survival. Studies using keratinocyte-specific Stat3-deficient mice have revealed that Stat3 plays an important role in skin homeostasis including keratinocyte migration, wound healing, and hair follicle growth. Use of both constitutive and inducible keratinocyte-specific Stat3-deficient mouse models has demonstrated that Stat3 is required for both the initiation and promotion stages of multistage skin carcinogenesis. Further studies using a transgenic mouse model with a gain of function mutant of Stat3 (Stat3C) expressed in the basal layer of the epidermis revealed a novel role for Stat3 in skin tumor progression. Studies using similar Stat3-deficient and gain-of-function mouse models have indicated its similar roles in ultraviolet B (UVB) radiation-mediated skin carcinogenesis. This paper summarizes the use of these various mouse models for studying the role and underlying mechanisms for the function of Stat3 in skin carcinogenesis. Given its significant role throughout the skin carcinogenesis process, Stat3 is an attractive target for skin cancer prevention and treatment.

## 1. Introduction

**Figure 1:**
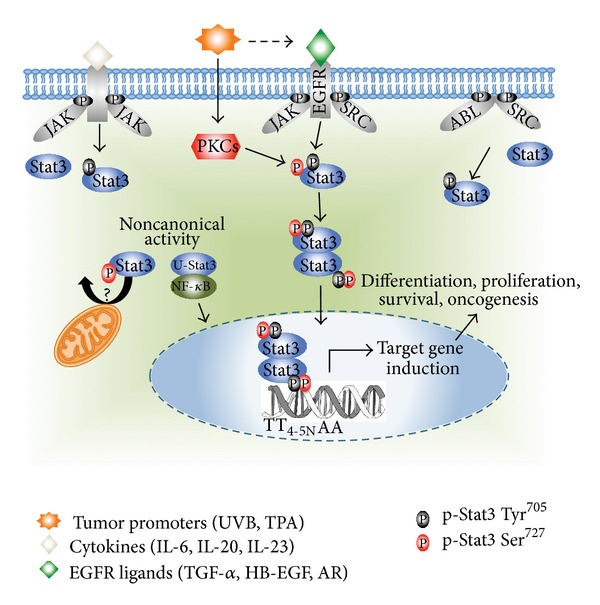
Pathways associated with Stat3 activation. Stat3 is activated downstream of receptor tyrosine kinases (e.g., EGFR), cytokine receptors via associated Janus family kinases (JAKs) (e.g., IL-6 receptor), and nonreceptor-associated tyrosine kinases (e.g., c-src). Tumor promoters such as TPA and UVB activate Stat3 in keratinocytes primarily via the EGFR. Activation of PKCs by tumor promoters leads to the processing of membrane-bound proforms of EGFR ligands such as heparin-binding EGF (HB-EGF) by matrix metalloproteinases (MMPs). In addition, PKCs associate with and phosphorylate Stat3 at Ser^727^, which is necessary for maximal Stat3 transcriptional activity. Furthermore, transcriptional induction of cytokines and EGF ligands can lead to autocrine stimulation and sustained Stat3 phosphorylation. After phosphorylation, STAT3 dimerizes and translocates to the nucleus, where Stat3 dimers directly regulate gene expression of transcriptional targets including Bcl-x_L_, cyclin D1, c-myc, Twist and Survivin. STAT3-mediated regulation of target gene expression is involved in various cellular functions including cell differentiation, proliferation, survival, and oncogenesis. Stat3 can also act through noncanonical signaling pathways. In this regard, unphosphorylated Stat3 (U-Stat3) can drive gene expression of a subset of genes that are different from p-Stat3 dimers in an NF-*κ*B-dependent and independent manner. In addition, p-Stat3 Ser^727^ can translocate into the mitochondria and influence mitochondrial respiratory chain activity. These noncanonical Stat3 signaling pathways have protumorigenic roles in certain cell/tissue types; however their role in epithelial carcinogenesis has not been evaluated.

Signal transducers and activators of transcription (Stats) are proteins that are activated by extracellular signaling proteins, such as growth factors, cytokines and various peptides [1]. Stats can also be activated via nonreceptor tyrosine kinases (e.g., src and abl) [1]. Cell-surface-mediated receptor activation causes the phosphorylation of tyrosine kinases, such as Janus-associated-kinase (Jak), which provides docking sites for the src homology (SH2) domain, enabling the binding and subsequent phosphorylation of Stats. The reciprocal interaction between the SH2 domains of two phosphorylated Stat monomers results in the formation of a functional Stat dimer. By this process, the Stat proteins are recruited to Jaks and are phosphorylated at their critical tyrosine residues. The phosphorylated Stats dimerize, translocate to the nucleus, and drive transcription of their target genes (Figure 1) [2].

While activation of Stats downstream of ligand-induced receptor activation is linked to differentiation and growth regulation functions, constitutive activation of Stats is often associated with deregulated cell growth [1]. There are seven different Stat proteins, Stat1 through Stat6, including two isoforms of Stat5 (Stat5a and Stat5b). Stat1, Stat3, Stat4, Stat5a, and Stat5b all form homodimers. In addition, Stat1, Stat2, and Stat3 can form heterodimers. Phenotypic analysis of genetically targeted mouse models for individual Stat genes has aided in delineating their biological roles. Interestingly, of all the Stat proteins only deletion of Stat3 leads to embryonic lethality [3]. Stat3 was originally identified as an IL-6-dependent transcription factor that promotes acute phase gene expression [4, 5]. However, subsequent studies have shown Stat3 activation by various cytokines, growth factors, and hormones [1]. In addition to its role in numerous cellular functions, there is strong evidence correlating Stat3 activation and cancer. Stat3 is found constitutively activated in cells transformed by the oncogenes v-Src and v-Abl, as well as in various human cancers, including hematologic, pancreas, breast, head and neck, and prostate cancer [6, 7]. Although there is substantial data in the literature on the protumorigenic effects of Stat3, there have been reports that activation of Stat3 can have an opposite, tumor-suppressive role (e.g., PTEN wt versus null gliomas) [8] and that activated Stat3 is associated with better prognosis in leiomyosarcoma and human papillary thyroid carcinoma [9, 10].

The mouse skin model of multistage carcinogenesis has been used for over 60 years and is one of the most well-established *in vivo* models for studying the stepwise and chronological development of epithelial tumors [11, 12]. Multistage chemical carcinogenesis in this model can be subdivided into three stages: initiation, promotion, and progression. The initiation step involves application of a subcarcinogenic dose of a carcinogen such as 7,12-dimethylbenz[a]anthracene (DMBA), which induces mutations in gene(s) through metabolism to reactive diol-epoxide metabolites and their subsequent covalent binding to DNA forming DNA adducts. The Ha-ras gene is a primary target of DMBA in this model and is routinely found mutated at codon 61 (A to T mutation) in tumors generated by initiation with polycyclic aromatic hydrocarbon [11]. Subsequently, the process of tumor promotion is accomplished by the repeated application of a tumor-promoting agent, most commonly the phorbol ester, 12-*O*-tetradecanoylphorbol-13-acetate (TPA). TPA treatment induces epidermal proliferation and clonal expansion of initiated cells. Lastly, the tumor progression stage occurs stochastically and leads to the conversion of skin papillomas to squamous cell carcinomas (SCCs) [11–14]. In this model of skin carcinogenesis, Stat3 is activated very early in the epidermis following treatment with different classes of tumor promoters, including TPA, okadaic acid, and chrysarobin [15]. In addition, Stat3 is upregulated and constitutively activated in skin papillomas and SCCs generated by the two-stage protocol. The primary mechanism for activation of Stat3 in mouse keratinocytes exposed to tumor promoters is through activation of epidermal growth factor receptor (EGFR) (Figure 1) [15], although other pathways may also contribute to its activation during tumor promotion. Given the unique embryonic lethal phenotype of Stat3 targeting, tissue-specific knockout and inducible knockout strategies using the Cre-*loxP* system have been used to elucidate the role(s) of Stat3 in skin biology and skin carcinogenesis [16]. Loss-of-function studies have been complimented by a Stat3 mouse model expressing a constitutively activated Stat3 protein (Stat3C) under the control of the keratin 5 promoter (K5.Stat3C transgenic mice). Studies from this comprehensive set of skin-specific knockout and transgenic Stat3 mouse models have shown that Stat3 plays a major role in skin carcinogenesis. Herein, we review the use of these mouse models and the insights gained regarding the role of Stat3 in skin carcinogenesis. 

## 2. Skin-Specific Deletion of Stat3 Reveals Roles in Wound Healing and Hair Cycle

Due to the embryonic lethality of Stat3^−/−^ mice, mice with conditional deletion of Stat3 in the skin were generated by crossing Stat3^flox/−^ mice with a transgenic line expressing Cre recombinase under the control of the human keratin 5 promoter (K5.*Cre*) [17, 18]. The K5 promoter drives gene expression in the basal cell layer of the epidermis and follicular keratinocytes [19]. Thus, expression of Cre recombinase under control of K5 allowed for deletion of the Stat3 gene throughout the epidermis and the outer root sheath of hair follicles [18, 19]. Unlike Stat3^−/−^ mice, K5.*Cre*  ×  Stat3^flox/−^ mice were viable, developed normally, and exhibited normal skin at a young age. However, Stat3 disruption in these mice impaired keratinocyte migration during the wound healing process both *in vivo* and *in vitro*. In addition, Stat3 loss compromised mainly the second anagen phase of the hair cycle [17]. Thus, older mice developed a sparse hair coat and also developed spontaneous skin ulcers due to impaired wound healing. From these studies it was concluded that Stat3 was not involved in skin morphogenesis but that it plays a significant role in skin remodeling through its effects on wound healing and the hair cycle [17, 20].

## 3. Skin-Specific Deletion of Stat3 Reveals Roles in both the Initiation and Promotion Stages of Two-Stage Chemical Carcinogenesis

Various forms of skin wounding are known to promote skin tumors in mice [11]. As mentioned above, studies using mice with a keratinocyte-specific deletion of Stat3 revealed a significant role for Stat3 in skin wound healing. Therefore, further studies were conducted to examine the possible role of Stat3 in skin carcinogenesis. For these studies, mice containing both floxed Stat3 alleles (i.e., Stat3^flox/flox^ mice) were used to reduce the severity of the wounding defect seen in mice harboring one floxed allele and one null allele (i.e., Stat3^flox/−^ mice) in the original studies [17]. Stat3^flox/flox^ mice were crossed with K5.Cre mice to produce an epidermis-specific deficiency Stat3 [21]. Thus, use of this skin-specific Stat3-deficient mouse model demonstrated that Stat3 was absolutely required for development of skin tumors using the two-stage DMBA-TPA protocol. In this regard, loss of Stat3 completely suppressed the development of skin papillomas. Further studies using these mice revealed that Stat3 deficiency sensitized keratinocytes to DMBA-induced apoptosis both *in vivo* and *in vitro *[21]. Interestingly, Stat3-deficient keratinocytes that underwent apoptosis after DMBA treatment *in vivo* were localized in the bulge region adjacent to label-retaining cells (LRCs) [21]. The proximity of DMBA-sensitive cells to LRCs raised the possibility that the loss of Stat3 induced a loss of initiated keratinocyte stems cells in this model (discussed in more detail below). These data suggested that Stat3 played a role during the initiation stage of skin carcinogenesis through its ability to regulate genes involved in keratinocyte survival during the process of tumor initiation.

The repeated treatment with TPA after initiation is used to induce epidermal proliferation and clonal expansion of initiated cells that harbor Ha-ras mutations, which ultimately leads to development of premalignant papillomas [11, 12]. Loss of Stat3 in K5.Cre  ×  Stat3^flox/flox^ mice resulted in a significant reduction of epidermal hyperproliferation (assessed by bromodeoxyuridine (BrdU) labeling index) compared to control littermates following TPA treatment [15, 21]. Mechanistic studies showed that recovery of cell cycle regulatory proteins cyclin D1 and cyclin E was delayed and c-myc expression was persistently downregulated in the epidermis of K5.Cre  ×  Stat3^flox/flox^ mice after topical treatment with TPA in comparison to control mice. Thus, constitutive deletion of Stat3 in the basal layer of epidermis inhibited TPA-induced epidermal hyperproliferation during tumor promotion. Additional studies investigating the role of Stat3 in clonal expansion of initiated cells during promotion were conducted using the TG.AC mouse model. TG.AC transgenic mice express a fusion protein of the activated v-Ha-Ras oncogene and the mouse zeta-globin gene [29]. In this model expression of activated v-Ha-Ras replaces the initiation step (DMBA treatment) of the two-stage chemical carcinogenesis protocol. Promotion in TG.AC mice with TPA results in development of multiple papillomas that progress to SCCs [29]. Inhibition of Stat3 using an oligonucleotide decoy targeting Stat3 inhibited TPA-induced papilloma formation in TG.AC mice, confirming that Stat3 is required for the clonal expansion of initiated cells during the promotion phase of two-stage skin carcinogenesis [21]. Moreover, intratumoral injection of Stat3 decoy caused regression in preexisting skin papillomas [21]. Collectively, these studies using K5.Cre  ×  Stat3^flox/flox^ mice indicated that Stat3 is required for survival of keratinocytes that have accumulated DNA damage during initiation with DMBA and that initiated keratinocytes harboring Ha-ras mutations require Stat3 for proliferation and clonal expansion during tumor promotion with TPA.

## 4. Inducible Stat3 Deficiency Using K5. CreER^T2^  ×  Stat3^flox/flox^  Mice Directly Confirms a Role for Stat3 in Both the Initiation and Promotion Stages of Skin Carcinogenesis

While the studies using K5.Cre  ×  Stat3^flox/flox^ mice provided strong evidence that Stat3 was involved in both the initiation and promotion stages of skin carcinogenesis, more direct evidence was obtained using an inducible system where Stat3 could be deleted in a temporal manner. Thus, intercross of Stat3^flox/flox^ mice with a transgenic mouse expressing a tamoxifen inducible *Cre* (i.e., Cre-ER^T2^) gene under the control of the K5 promoter (K5.CreER^T2^ mice) provided a temporally controlled and inducible epidermis-specific Stat3-deficient mouse model [24, 30]. Using this mouse model, temporal disruption of Stat3 at the time of initiation resulted in an increased number of apoptotic cells following DMBA treatment [24]. In a two-stage carcinogenesis experiment, tamoxifen treatment prior to DMBA treatment of inducible Stat3-deficient mice significantly delayed tumor onset and reduced the number of papillomas per mouse [24]. Similarly, inducible deletion of Stat3 prior to each TPA treatment during the tumor promotion stage delayed tumor onset and tumor multiplicity. Mechanistic studies confirmed that deletion of Stat3 using this inducible system led to reduced levels of survival proteins such as Bcl-x_L_ and S-phase proteins such as cyclin D1, cyclin E, and c-myc supporting the earlier observations using K5.Cre  ×  Stat3^flox/flox^ mice. The availability of an inducible Stat3 knockout model also allowed deletion of Stat3 in skin papillomas generated by the DMBA-TPA protocol. In this regard, deletion of Stat3 in skin papillomas by i.p. injection of tamoxifen inhibited subsequent growth of these tumors. Collectively, these studies using an inducible Stat3 knockout system provided direct evidence for a role of Stat3 in both the initiation and promotion stages of skin carcinogenesis. Furthermore, these studies showed that deletion of Stat3 reduced the levels of both survival proteins and cell cycle proteins involved in G1 to S-phase transition. Finally, the use of these models demonstrated the requirement for Stat3 activation for continued growth of skin papillomas.

## 5. Stat3C Transgenic Mice Reveal a Novel Role for Stat3 in Skin Tumor Progression

Constitutive activation of Stat3 is observed in a variety of human tumors [6, 7] as noted above. This persistent activation can be recapitulated experimentally by substituting residues A661 and N663 for cysteine residues, allowing for cysteine-cysteine sulfhydryl bonds between Stat3 monomers and the formation of Stat3 homodimers without the phosphorylation of Tyr^705^ [31]. This form of Stat3, referred to as Stat3C, was initially shown to transform mouse and rat fibroblasts as demonstrated by anchorage-independent growth in soft agar and formation of tumors when these cells were injected into nude mice [31]. Further study of the role of Stat3 in skin carcinogenesis was facilitated by the generation of mice that express this constitutively active/dimerized form of Stat3 targeted to the proliferative compartment of epidermis using the bovine K5 promoter (i.e., K5.Stat3C transgenic mice) [27]. K5.Stat3C transgenic mice did not develop spontaneous tumors but did exhibit a mild hyperproliferative epidermis and developed spontaneous psoriatic skin lesions with age [28]. In addition, K5.Stat3C mice showed an increased BrdU labeling index after TPA treatment compared to nontransgenic littermates. Expression of Stat3C in the basal compartment of the epidermis significantly protected keratinocytes from DMBA-induced apoptosis [27]. In a DMBA-TPA skin carcinogenesis protocol, K5.Stat3C mice developed skin tumors in greater number and with a shortened latency compared to nontransgenic littermates. Notably, 100% of skin tumors that developed in K5.Stat3C transgenic mice bypassed the premalignant (papilloma) stage and initially developed as carcinoma *in situ*. Histological and immunohistochemical analyses revealed that these tumors were highly vascularized and poorly differentiated, and invasion into surrounding dermal/mesenchymal tissue was observed at a very early stage. Expression of K10, filaggrin, and E-cadherin was completely lost in skin tumors from K5.Stat3C transgenic mice by 20 weeks [27]. Thus, expression of a constitutively active form of Stat3 significantly increased the rate of tumor progression in this model system. This effect of Stat3 was associated with increased expression of Twist, a transcription factor known to regulate genes involved in epithelial-mesenchymal transition (EMT) [27, 32]. Thus, use of this unique mouse model led to the discovery that Stat3 plays an important role not only in the initiation and promotion stages of skin carcinogenesis but also during the progression stage.

## 6. Deletion of Stat3 in Bulge Region Keratinocyte Stem Cells

Keratinocyte stem cells (KSCs) located in the bulge region of hair follicles are self-renewing cells that provide transit-amplifying cells necessary for hair regrowth and skin homeostasis [33]. In addition, KSCs in the bulge region are believed to be target cells for tumor development in two-stage chemical carcinogenesis of mouse skin [34, 35]. The K15 promoter has been reported to be specifically active in the bulge region of the murine hair follicle and has been used to characterize KSCs in the bulge region. For loss-of-function studies of bulge region KSCs, Morris [35] generated K15.CrePR1 transgenic mice. Cre-PR1 is a fusion protein that consists of Cre recombinase and a truncated form of the progesterone receptor that binds to the progesterone antagonist RU486 but not to endogenous progesterone [35, 36]. To further investigate the role of Stat3 in bulge region keratinocytes during multistage skin carcinogenesis, Dae et al. [25] utilized the K15.CrePR1 transgenic mouse model in combination with Stat3^flox/flox^ mice. Using this inducible model, Stat3 deletion at the time of initiation in bulge region keratinocytes led to a significant reduction in tumor incidence and multiplicity (~80% reduction in papilloma formation). The K15.CrePR1 inducible system is not 100% efficient and the small number of papillomas obtained from these knockout mice stained positive for Stat3. This data indicated that Stat3 is absolutely necessary for tumor development, since the remaining Stat3-positive KSCs were selected during tumor promotion. In addition, DMBA treatment led to a significant increase in the number of apoptotic keratinocytes in the bulge region of the knockout mice. FACS analysis showed that there was a reduction in the percentage of bulge region KSCs that were positive for CD34 and *α*6-integrin in the knockout mice compared to the control mice 24 hours after DMBA treatment. Furthermore, the *α*6+CD34+ population from K15.CrePR1  ×  Stat3^flox/flox^ mice showed a reduction of the signature Ha-ras codon 61 A^182^ to T mutation induced by topical application of DMBA [25]. Hence, Stat3 status influenced survival of DNA-damaged KSCs, since the absence of Stat3 led to increased apoptosis of bulge region KSCs following treatment with DMBA ultimately leading to reduced number of mutated cells available for clonal expansion during tumor promotion [25]. These data suggested that Stat3 plays an important role in the behavior of bulge region KSCs during the initiation step of skin tumor development by the two-stage chemical carcinogenesis protocol. 

## 7. Stat3 in UVB-Induced Skin Carcinogenesis

The availability of both loss-of-function and gain-of-function mouse models for Stat3 in skin keratinocytes facilitated further study of this important molecule in skin carcinogenesis mediated by ultraviolet B (UVB) exposure. UV radiation, and in particular UVB exposure, is the major risk factor for nonmelanoma skin cancer in humans [37]. Following exposure to UVB, the level of phosphorylated Stat3 (p-Stat3) is initially decreased, followed by a significant increase at later time points in the mouse epidermis. The levels of Stat3 target genes, such as cyclin D1, Bcl-x_L_, and c-Myc, followed the changes in activated Stat3 in response to UVB irradiation [38]. Epidermis-specific Stat3-deficient mice were found to be very sensitive to UVB radiation as revealed by a higher number of sunburned and apoptotic cells following irradiation with UVB [22]. On the other hand, the epidermis of K5.Stat3C mice was significantly resistant to UVB-induced apoptosis [23]. Furthermore, additional studies showed that protection against UVB-induced apoptosis in Stat3C transgenic mice was not due to impaired DNA damage response. Instead, the status of Stat3 influenced the survival of cells containing UVB-induced DNA photoproducts, including those cells located in the bulge region of the hair follicles through regulation of antiapoptotic genes such as Bcl-x_L_ [23, 26]. In line with these observations, overexpression of Stat3C in K5.Stat3C mice enhanced UVB-induced skin carcinogenesis (both incidence and tumor multiplicity) compared to the wild-type controls [23]. In contrast, Stat3-deficient mice were resistant to UVB skin carcinogenesis compared to wild-type controls [23]. Thus, based on these studies Stat3 appears to play a strikingly similar role in both chemical and UVB-mediated skin carcinogenesis.

## 8. Studies Using Other Mouse Models Support an Important Role of Stat3 in Skin Carcinogenesis

As noted above Bcl-x_L_ is one of several antiapoptotic proteins regulated by Stat3 [39]. Deletion of Stat3 in keratinocytes leads to a concomitant and dramatic reduction in levels of Bcl-x_L_ [24]. To study the functional role of Bcl-x_L_ in skin carcinogenesis, skin-specific Bc-x_L_-deficient mice were generated. In this model, Bcl-x_L_ expression is disrupted in the basal compartment of mouse epidermis using the bovine K5 promoter to drive expression of Cre recombinase (i.e., K5.Cre × Bcl-x_L_^flox/flox^ mice). A significant increase in apoptosis induced by either UVB irradiation or DMBA treatment was observed in the epidermis of Bcl-x_L_-deficient mice. Furthermore, an increase in apoptotic cells was noted in hair follicle keratinocytes, including those located in the bulge region. Cell proliferation was not affected by Bcl-x_L_ deficiency following exposure to either UVB or TPA. Bcl-x_L_-deficient mice were more resistant than wild-type controls to skin tumor development with delayed onset and reduced number of tumors using either UVB complete carcinogenesis or the DMBA/TPA two-stage regimen. Moreover, Bcl-2, Mcl-1, and survivin protein levels were increased in the epidermis of Bcl-x_L_-deficient mice in the absence of stimuli. Furthermore, levels of these antiapoptotic proteins were also high in skin tumors from Bcl-x_L_-deficient mice that developed in response to either UVB or two-stage carcinogenesis protocols. Collectively, these studies demonstrated that Bcl-x_L_ plays a role early in skin carcinogenesis through its antiapoptotic functions to enhance survival of keratinocytes, including bulge region KSCs, following DNA damage. These data also demonstrated that one of the antiapoptotic genes known to be regulated by Stat3 was likely mediating at least some of its action during the initiation stage of multistage skin carcinogenesis. However, deletion of Bcl-x_L_ did not fully recapitulate the actions of Stat3 deletion during initiation indicating that other Stat3-regulated genes are also likely involved in the action of Stat3.

It is also noteworthy that Stat3 function appears to be necessary for epidermal hyperplasia and susceptibility to skin tumor formation in other transgenic mouse models. In this regard, Stat3 has been implicated in proteins kinase c epsilon (PKC*ε*) mediated UV skin carcinogenesis. Stat3 Tyr^705^ and Ser^727^ phosphorylation is increased in K5.  PKC*ε* transgenic epidermis after UV irradiation compared to controls. Like Stat3C, overexpression of PKC*ε* in mouse skin (K5.PKC*ε*) inhibited apoptosis, promoted cell survival, and induced development of SCCs in UVB skin carcinogenesis experiments [40–42]. These studies show an interaction between PKC*ε* and Stat3 that leads to Stat3 activation by phosphorylation at Ser^727^. Phosphorylation at Ser^727^ is necessary for full transcriptional activity of Stat3 *in vivo* and may aid in the development of SCCs in K5.PKC*ε* transgenic mice [43]. Collectively, these studies demonstrate that Stat3 plays a critical role in the development of UVB-induced skin tumors through its effects on both survival and proliferation of keratinocytes [23]. 

 In other studies, overexpression of human papillomavirus 8 (HPV8) in mouse skin using the K14 promoter (i.e., K14.HPV8 transgenic mice) leads to epidermal hyperplasia and the development of spontaneous SCCs [44]. These tumors exhibited an increased level of p-Stat3 Tyr^705^ [44]. In a two-stage carcinogenesis bioassay, loss of a single Stat3 allele reduced HPV8-mediated epidermal hyperplasia and skin tumorigenesis [44]. Loss of Stat3 also produced similar effects in a mouse model of basal cell carcinoma [45]. In this model, SmoM2 expression in the basal epidermal layer was also driven by the K14 promoter (i.e., K14.SmoM2 transgenic mice). Epidermal-specific knockout of Stat3 in the K14.SmoM2 transgenic mice significantly reduced SmoM2-mediated epidermal hyperplasia and tumor development [45]. These studies further demonstrate the importance of Stat3 in skin carcinogenesis.

## 9. Stat3 and Human Skin Cancer

Activation of Stat3 appears to play an important role in the development of human nonmelanoma skin cancer. Stat3 is found constitutively activated in UVB-induced SCCs from both mouse and human skin [22]. Increased p-Stat3 expression in human SCCs and basal cell carcinomas (BCCs) in comparison to normal skin has been observed in various retrospective studies [46–49]. This increased expression of p-Stat3 is inversely associated with cellular differentiation, with expression in poorly differentiated SCCs being significantly higher than in well-differentiated SCCs [46, 47]. A positive correlation between p-Stat3 expression and depth of tumor invasion, but not tumor size, was also observed [46, 47]. Furthermore, like SCCs derived from K5.Stat3C transgenic mice, human SCCs display a negative correlation between the expression of p-Stat3 and E-cadherin [27, 46]. Stat3-mediated downregulation of E-cadherin, in conjunction with Stat3-mediated induction of twist as observed in K5.Stat3C-derived SCCs, may promote EMT and contribute to the metastatic potential of SCCs. These data suggest that Stat3 plays an important role in the development and progression of human SCCs.

Head and neck cancers are a group of biologically similar cancers that stem from the lip, oral cavity, nasal cavity, paranasal sinuses, pharynx, and larynx. In this group of cancers, it is estimated that 90% are SCCs or head and neck squamous cell carcinoma (HNSCC), which originate from the mucosal lining (epithelium) in these tissues [50]. HNSCCs are very similar histologically to SCCs of the skin and both share a number of similar molecular alterations. In particular, as observed in cutaneous SCCs, preclinical and clinical studies have implicated Stat3 in the development and progression of HNSCCs [51–54]. In head and neck tumor tissue, Stat3 is found upregulated and constitutively activated (phosphorylated) and has a positive correlation with poor prognosis [51, 54]. Preclinical studies have shown that HNSCC cell lines stably transfected with a constitutively active STAT3 construct expressed elevated levels of STAT3 target genes, including Bcl-x_L_ and cyclin D1, leading to increased proliferation *in vitro* and more rapid tumor growth rates *in vivo *[55]. In addition, targeting Stat3 increases tumor cell apoptosis and decreased Bcl-x_L_ expression in a head and neck xenograft model [53]. In addition, reducing Stat3 activity by targeting upstream proteins has shown promise in HNSCC preclinical and clinical studies. In this regard, inhibiting EGFR-Stat3 pathway in 4-nitroquinoline-1-oxide (4-NQO-) induced murine model of oral carcinogenesis by erlotinib, a small molecule inhibitor of EGFR, inhibited development of preneoplastic lesions and oral tumors by approximately 70% with a concomitant decrease of Stat3 levels in erlotinib-treated mice [56]. Similarly, primary human oral cavity squamous cell cancers showed reduced levels of both EGFR and p-Stat3 after treatment with erlotinib compared to pretreated paired tissue [57]. Moreover, combined molecular targeting of Stat3 sensitizes cells to radiotherapy and small molecule chemotherapeutic agents *in vitro* [58, 59]. Together, these data have provided the basis for targeting Stat3 in HNSCC in a clinical setting (discussed below). 

## 10. Perspectives and Future Directions

**Figure 2:**
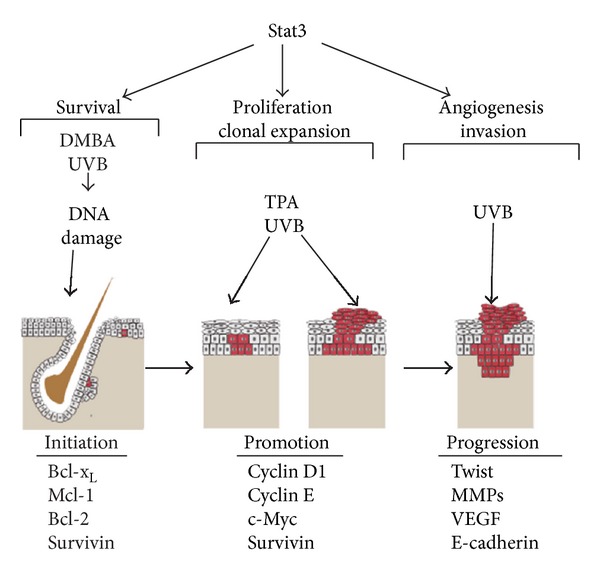
From studies in loss-of-function and gain-of-function mouse models, Stat3 has been shown to have a critical role in all three stages of skin carcinogenesis whether induced by chemicals (using the standard DMBA-TPA protocol) or by complete carcinogenesis with UVB. During the initiation stage, Stat3 aids in the survival of DNA-damaged keratinocyte stem cells (bulge region and possibly those in the interfollicular epidermis) induced by DMBA or UVB-irradiation by upregulation of prosurvival proteins such as bcl-x_L_, bcl-2, mcl-1, and survivin. Clonal expansion of initiated cells (promotion) is carried out through repeated treatment with TPA or UVB. In this stage, Stat3-mediated induction of cell cycle regulatory proteins (e.g., cyclin D1, cyclin E, c-myc, and survivin) is necessary for keratinocyte proliferation, epidermal hyperplasia, and development of papillomas/premalignant lesions. Progression or conversion of papillomas to SCCs is denoted as a downward invading lesion that traverses into the dermal compartment. Stat3 plays a role in the progression stage by regulating genes involved in angiogenesis and invasion (e.g., VEGF, MMPs, twist, E-cadherin).

Collectively, the comprehensive set of skin-specific gain- and loss-of-function mouse models described above have revealed that Stat3 plays a critical role in all three stages of skin carcinogenesis induced by either chemical exposure or UVB irradiation (see Table 1 for a listing of these mouse models). Stat3 is required for survival of DNA-damaged KSCs and the proliferation necessary for clonal expansion of initiated cells during tumor promotion. In addition, Stat3 also appears to have a role in driving malignant conversion of skin tumors. Although UVB is a complete carcinogen, it possesses both initiating and promoting activity that can be distinguished experimentally. From the available data, Stat3 appears to play a very similar mechanistic role during UVB-mediated skin carcinogenesis. Figure 2 summarizes our current state of knowledge regarding Stat3 function in skin carcinogenesis. 

The current data also suggest that targeting Stat3 activation may provide an effective strategy for both the prevention and treatment of skin cancer. However, targeting Stat3 directly has proven to be difficult and this molecule has largely seemed “undruggable” [60]. Many approaches have been taken to target Stat3 activity, including inhibition of upstream proteins such as receptor and nonreceptor tyrosine kinases, targeting of Stat3 SH2 domains to prevent phosphorylation/dimerization, inhibition of Stat3 DNA-binding activity, and inhibition of nuclear import [61]. Until recently, inhibition of upstream regulatory proteins has made the most progress in clinical trials [62]. Phase 0 (University of Pittsburgh, Pittsburgh, PA) and phase 1 clinical trials (MD Anderson Cancer Center, Houston, TX, USA and Otsuka Pharmaceuticals, Princeton, NJ, USA) evaluating Stat3 inhibitors have recently been completed. In addition, a phase 1 (Isis Pharmaceuticals, San Diego, CA, USA) and an observational clinical trial (New York University, New York, NY, USA) are in the recruiting stages. The completed phase 0 clinical trial evaluated the safety of an oligonucleotide decoy targeting Stat3 by intratumoral injection in patients with HNSCC [63]. Interestingly, a single intratumoral injection with a Stat3 decoy showed decreased Stat3 target gene expression of cyclin D1 and Bcl-x_L_ in HNSCC biopsies [63]. Moreover, by circularizing the 15-base pair oligonucleotide with two hexaethylene glycol linkages, Sen and colleagues were able to inhibit tumor growth in preclinical mouse xenografts via systemic administration [63]. These studies offer promise for expanded phase 1 clinical trials in HNSCC patients and a wide range of malignancies that are dependent on Stat3 activation, including nonmelanoma skin cancers. 

Recent evidence suggests a novel role for Stat3 in mitochondrial respiration, presumably via its interaction with electron transport chain components [64] and see again Figure 1. Mitochondrial localized Stat3 (referred to as mitoStat3) is necessary for Ha-ras-mediated transformation of mouse embryonic fibroblasts independent of Stat3 nuclear activity or tyrosine phosphorylation [65]. In addition, unphosphorylated Stat3 (U-Stat3), which was previously thought to be an inactive protein, has recently been shown to regulate gene transcription through a mechanism distinct from that of tyrosine-phosphorylated Stat3 dimers [66–68]. U-Stat3 has been shown to interact with nuclear factor-*κ*B (NF-*κ*B) (Figure 1) and regulate genes with *κ*B elements, but it can also induce a cohort of genes through an NF-*κ*B-independent mechanism [67]. Expression of U-Stat3 enhances hepatocellular carcinoma (HCC) formation in Ras-transformed p19^ARF−/−  ^ hepatocytes [67, 69]. Whether mitoStat3 or U-Stat3 plays a role in epithelial carcinogenesis is unclear at the present time. Future studies should also evaluate these potential mechanisms and will likely require the development of additional mouse models. In addition, targeting or exploiting these noncanonical activities associated with Stat3 for both prevention and treatment strategies may also be warranted.

## Figures and Tables

**Table 1:** Mouse models for evaluating Stat3 function in skin carcinogenesis.

Mouse model	Skin phenotype	Susceptibility to skin carcinogenesis	References
K5.Cre × Stat3^flox/−^	(i) Defective wound healing (ii) Defective hair cycle from 2nd anagen onward	Not tested	[17]
K5.Cre × Stat3^flox/flox^	No visible phenotype	Reduced susceptibility to both DMBA-TPA and UVB carcinogenesis	[21–23]
K5.CreER^T2^× Stat3^flox/flox^	No visible phenotype	Reduced susceptibility to both tumor initiation with DMBA and tumor promotion with TPA; UVB not tested	[24]
K15.CrePR1 × Stat3^flox/flox^	No visible phenotype	Reduced susceptibility to tumor initiation by DMBA; UVB not tested	[25]
K5.Cre × Bcl-x_L_^flox/flox^	No visible phenotype	Reduced susceptibility to both DMBA-TPA and UVB carcinogenesis	[26]
K5.Stat3C	(i) Enlarged blood vessels in skin at birth (ii) Sparse hair coat (iii) Increased skin vascularization in adult mice (iv) Hypervascularization in response to mild wounding (e.g., tape stripping) (v) Develop scaly, hyperkeratotic lesions on tail (psoriasis) (vi) No spontaneous skin tumors	Enhanced susceptibility to DMBA-TPA and UVB skin carcinogenesis Enhanced progression of skin tumors to SCCs	[22, 23, 27, 28]
